# Supplementary material for: Pyruvate Dehydrogenase Kinase 1 inhibition mediated oxidative phosphorylation enhancement in cartilage promotes osteoarthritis progression
Source: BMC Musculoskelet Disord. 2023 Jul 20;24:597. doi: 10.1186/s12891-023-06585-6 (PMC10357736; doi:10.1186/s12891-023-06585-6)
Supplement: Supplementary file 1 — Additional file 1. [file 12891_2023_6585_MOESM1_ESM.zip › KEGG.Gsea.1653622432464/KEGG_TIGHT_JUNCTION.html]

Details for gene set KEGG\_TIGHT\_JUNCTION[GSEA]

|  || Dataset | 55457\_collapsed\_to\_symbols.cls.cls #OA\_versus\_normal.cls.cls #OA\_versus\_normal\_repos |
| Phenotype | cls.cls#OA\_versus\_normal\_repos |
| Upregulated in class | normal |
| GeneSet | KEGG\_TIGHT\_JUNCTION |
| Enrichment Score (ES) | -0.31514373 |
| Normalized Enrichment Score (NES) | -1.4059042 |
| Nominal p-value | 0.018461538 |
| FDR q-value | 0.2066625 |
| FWER p-Value | 0.994 |
Table: GSEA Results Summary

  

Fig 1: Enrichment plot: KEGG\_TIGHT\_JUNCTION      
 Profile of the Running ES Score & Positions of GeneSet Members on the Rank Ordered List

  

| SYMBOL | TITLE | RANK IN GENE LIST | RANK METRIC SCORE | RUNNING ES | CORE ENRICHMENT || 1 | RRAS | RAS related [Source:HGNC Symbol;Acc:HGNC:10447] | 505 | 0.658 | -0.0204 | No |
| 2 | AKT1 | AKT serine/threonine kinase 1 [Source:HGNC Symbol;Acc:HGNC:391] | 1192 | 0.478 | -0.0602 | No |
| 3 | CDK4 | cyclin dependent kinase 4 [Source:HGNC Symbol;Acc:HGNC:1773] | 1375 | 0.447 | -0.0614 | No |
| 4 | MYH13 | myosin heavy chain 13 [Source:HGNC Symbol;Acc:HGNC:7571] | 1561 | 0.419 | -0.0638 | No |
| 5 | CLDN4 | claudin 4 [Source:HGNC Symbol;Acc:HGNC:2046] | 1716 | 0.395 | -0.0643 | No |
| 6 | CSNK2B | casein kinase 2 beta [Source:HGNC Symbol;Acc:HGNC:2460] | 1788 | 0.385 | -0.0587 | No |
| 7 | MAGI2 | "membrane associated guanylate kinase, WW and PDZ domain containing 2 [Source:HGNC Symbol;Acc:HGNC:18957]" | 1980 | 0.363 | -0.0631 | No |
| 8 | CTNNA3 | catenin alpha 3 [Source:HGNC Symbol;Acc:HGNC:2511] | 2030 | 0.358 | -0.0565 | No |
| 9 | PRKCD | protein kinase C delta [Source:HGNC Symbol;Acc:HGNC:9399] | 2237 | 0.335 | -0.0629 | No |
| 10 | MYH1 | myosin heavy chain 1 [Source:HGNC Symbol;Acc:HGNC:7567] | 2297 | 0.327 | -0.0580 | No |
| 11 | CASK | calcium/calmodulin dependent serine protein kinase [Source:HGNC Symbol;Acc:HGNC:1497] | 2316 | 0.324 | -0.0500 | No |
| 12 | MYL2 | myosin light chain 2 [Source:HGNC Symbol;Acc:HGNC:7583] | 2376 | 0.317 | -0.0454 | No |
| 13 | MYH2 | myosin heavy chain 2 [Source:HGNC Symbol;Acc:HGNC:7572] | 2459 | 0.307 | -0.0429 | No |
| 14 | ACTN2 | actinin alpha 2 [Source:HGNC Symbol;Acc:HGNC:164] | 2532 | 0.299 | -0.0399 | No |
| 15 | MYH6 | myosin heavy chain 6 [Source:HGNC Symbol;Acc:HGNC:7576] | 2555 | 0.297 | -0.0330 | No |
| 16 | MYL5 | myosin light chain 5 [Source:HGNC Symbol;Acc:HGNC:7586] | 2705 | 0.279 | -0.0365 | No |
| 17 | PRKCZ | protein kinase C zeta [Source:HGNC Symbol;Acc:HGNC:9412] | 2760 | 0.274 | -0.0328 | No |
| 18 | MYLPF | "myosin light chain, phosphorylatable, fast skeletal muscle [Source:HGNC Symbol;Acc:HGNC:29824]" | 2816 | 0.268 | -0.0293 | No |
| 19 | MYL12A | myosin light chain 12A [Source:HGNC Symbol;Acc:HGNC:16701] | 3039 | 0.246 | -0.0395 | No |
| 20 | CLDN1 | claudin 1 [Source:HGNC Symbol;Acc:HGNC:2032] | 3089 | 0.241 | -0.0364 | No |
| 21 | MYH8 | myosin heavy chain 8 [Source:HGNC Symbol;Acc:HGNC:7578] | 3119 | 0.238 | -0.0317 | No |
| 22 | MYH7 | myosin heavy chain 7 [Source:HGNC Symbol;Acc:HGNC:7577] | 3161 | 0.234 | -0.0281 | No |
| 23 | EXOC3 | exocyst complex component 3 [Source:HGNC Symbol;Acc:HGNC:30378] | 3583 | 0.195 | -0.0554 | No |
| 24 | PRKCQ | protein kinase C theta [Source:HGNC Symbol;Acc:HGNC:9410] | 3658 | 0.189 | -0.0557 | No |
| 25 | GNAI3 | G protein subunit alpha i3 [Source:HGNC Symbol;Acc:HGNC:4387] | 3694 | 0.186 | -0.0530 | No |
| 26 | PRKCA | protein kinase C alpha [Source:HGNC Symbol;Acc:HGNC:9393] | 3728 | 0.183 | -0.0503 | No |
| 27 | PALS1 | "protein associated with LIN7 1, MAGUK family member [Source:HGNC Symbol;Acc:HGNC:18669]" | 3787 | 0.178 | -0.0497 | No |
| 28 | CTNNA1 | catenin alpha 1 [Source:HGNC Symbol;Acc:HGNC:2509] | 4003 | 0.156 | -0.0620 | No |
| 29 | MYL10 | myosin light chain 10 [Source:HGNC Symbol;Acc:HGNC:29825] | 4125 | 0.144 | -0.0672 | No |
| 30 | PPP2CA | protein phosphatase 2 catalytic subunit alpha [Source:HGNC Symbol;Acc:HGNC:9299] | 4146 | 0.143 | -0.0647 | No |
| 31 | PPP2R2D | protein phosphatase 2 regulatory subunit Bdelta [Source:HGNC Symbol;Acc:HGNC:23732] | 4152 | 0.142 | -0.0609 | No |
| 32 | MYH10 | myosin heavy chain 10 [Source:HGNC Symbol;Acc:HGNC:7568] | 4169 | 0.141 | -0.0581 | No |
| 33 | LLGL2 | LLGL scribble cell polarity complex component 2 [Source:HGNC Symbol;Acc:HGNC:6629] | 4604 | 0.104 | -0.0890 | No |
| 34 | MYH4 | myosin heavy chain 4 [Source:HGNC Symbol;Acc:HGNC:7574] | 4702 | 0.096 | -0.0938 | No |
| 35 | MYL9 | myosin light chain 9 [Source:HGNC Symbol;Acc:HGNC:15754] | 4939 | 0.077 | -0.1101 | No |
| 36 | MYH7B | myosin heavy chain 7B [Source:HGNC Symbol;Acc:HGNC:15906] | 5024 | 0.071 | -0.1146 | No |
| 37 | SPTAN1 | "spectrin alpha, non-erythrocytic 1 [Source:HGNC Symbol;Acc:HGNC:11273]" | 5131 | 0.062 | -0.1211 | No |
| 38 | MYH11 | myosin heavy chain 11 [Source:HGNC Symbol;Acc:HGNC:7569] | 5211 | 0.056 | -0.1256 | No |
| 39 | PPP2R2B | protein phosphatase 2 regulatory subunit Bbeta [Source:HGNC Symbol;Acc:HGNC:9305] | 5292 | 0.048 | -0.1305 | No |
| 40 | CLDN10 | claudin 10 [Source:HGNC Symbol;Acc:HGNC:2033] | 5479 | 0.034 | -0.1441 | No |
| 41 | JAM2 | junctional adhesion molecule 2 [Source:HGNC Symbol;Acc:HGNC:14686] | 5602 | 0.024 | -0.1529 | No |
| 42 | EPB41 | erythrocyte membrane protein band 4.1 [Source:HGNC Symbol;Acc:HGNC:3377] | 5841 | 0.006 | -0.1714 | No |
| 43 | MYH15 | myosin heavy chain 15 [Source:HGNC Symbol;Acc:HGNC:31073] | 5874 | 0.004 | -0.1738 | No |
| 44 | PRKCE | protein kinase C epsilon [Source:HGNC Symbol;Acc:HGNC:9401] | 5926 | 0.000 | -0.1777 | No |
| 45 | EPB41L3 | erythrocyte membrane protein band 4.1 like 3 [Source:HGNC Symbol;Acc:HGNC:3380] | 5927 | 0.000 | -0.1777 | No |
| 46 | CLDN17 | claudin 17 [Source:HGNC Symbol;Acc:HGNC:2038] | 5931 | 0.000 | -0.1780 | No |
| 47 | PPP2R2A | protein phosphatase 2 regulatory subunit Balpha [Source:HGNC Symbol;Acc:HGNC:9304] | 5946 | -0.001 | -0.1790 | No |
| 48 | CSNK2A2 | casein kinase 2 alpha 2 [Source:HGNC Symbol;Acc:HGNC:2459] | 6105 | -0.013 | -0.1910 | No |
| 49 | PRKCB | protein kinase C beta [Source:HGNC Symbol;Acc:HGNC:9395] | 6453 | -0.039 | -0.2170 | No |
| 50 | LLGL1 | LLGL scribble cell polarity complex component 1 [Source:HGNC Symbol;Acc:HGNC:6628] | 6528 | -0.044 | -0.2215 | No |
| 51 | PARD6B | par-6 family cell polarity regulator beta [Source:HGNC Symbol;Acc:HGNC:16245] | 6688 | -0.055 | -0.2324 | No |
| 52 | PATJ | PATJ crumbs cell polarity complex component [Source:HGNC Symbol;Acc:HGNC:28881] | 6795 | -0.062 | -0.2389 | No |
| 53 | SRC | "SRC proto-oncogene, non-receptor tyrosine kinase [Source:HGNC Symbol;Acc:HGNC:11283]" | 6940 | -0.075 | -0.2479 | No |
| 54 | AFDN | "afadin, adherens junction formation factor [Source:HGNC Symbol;Acc:HGNC:7137]" | 7077 | -0.086 | -0.2561 | No |
| 55 | MYL7 | myosin light chain 7 [Source:HGNC Symbol;Acc:HGNC:21719] | 7137 | -0.091 | -0.2580 | No |
| 56 | PRKCH | protein kinase C eta [Source:HGNC Symbol;Acc:HGNC:9403] | 7141 | -0.092 | -0.2556 | No |
| 57 | PPP2CB | protein phosphatase 2 catalytic subunit beta [Source:HGNC Symbol;Acc:HGNC:9300] | 7265 | -0.100 | -0.2623 | No |
| 58 | NRAS | "NRAS proto-oncogene, GTPase [Source:HGNC Symbol;Acc:HGNC:7989]" | 7300 | -0.102 | -0.2620 | No |
| 59 | MPDZ | multiple PDZ domain crumbs cell polarity complex component [Source:HGNC Symbol;Acc:HGNC:7208] | 7338 | -0.105 | -0.2619 | No |
| 60 | CLDN8 | claudin 8 [Source:HGNC Symbol;Acc:HGNC:2050] | 7517 | -0.120 | -0.2723 | No |
| 61 | ACTN3 | actinin alpha 3 [Source:HGNC Symbol;Acc:HGNC:165] | 7557 | -0.124 | -0.2718 | No |
| 62 | HCLS1 | hematopoietic cell-specific Lyn substrate 1 [Source:HGNC Symbol;Acc:HGNC:4844] | 7653 | -0.131 | -0.2754 | No |
| 63 | TJAP1 | tight junction associated protein 1 [Source:HGNC Symbol;Acc:HGNC:17949] | 7694 | -0.134 | -0.2746 | No |
| 64 | EPB41L1 | erythrocyte membrane protein band 4.1 like 1 [Source:HGNC Symbol;Acc:HGNC:3378] | 7704 | -0.134 | -0.2714 | No |
| 65 | CLDN6 | claudin 6 [Source:HGNC Symbol;Acc:HGNC:2048] | 7883 | -0.147 | -0.2811 | No |
| 66 | EPB41L2 | erythrocyte membrane protein band 4.1 like 2 [Source:HGNC Symbol;Acc:HGNC:3379] | 7934 | -0.150 | -0.2807 | No |
| 67 | HRAS | "HRas proto-oncogene, GTPase [Source:HGNC Symbol;Acc:HGNC:5173]" | 7977 | -0.153 | -0.2795 | No |
| 68 | AKT3 | AKT serine/threonine kinase 3 [Source:HGNC Symbol;Acc:HGNC:393] | 8008 | -0.156 | -0.2773 | No |
| 69 | MYH14 | myosin heavy chain 14 [Source:HGNC Symbol;Acc:HGNC:23212] | 8032 | -0.158 | -0.2746 | No |
| 70 | GNAI1 | G protein subunit alpha i1 [Source:HGNC Symbol;Acc:HGNC:4384] | 8110 | -0.164 | -0.2758 | No |
| 71 | MYH3 | myosin heavy chain 3 [Source:HGNC Symbol;Acc:HGNC:7573] | 8258 | -0.176 | -0.2822 | No |
| 72 | RAB3B | "RAB3B, member RAS oncogene family [Source:HGNC Symbol;Acc:HGNC:9778]" | 8326 | -0.180 | -0.2822 | No |
| 73 | ACTN1 | actinin alpha 1 [Source:HGNC Symbol;Acc:HGNC:163] | 8385 | -0.184 | -0.2814 | No |
| 74 | MYH9 | myosin heavy chain 9 [Source:HGNC Symbol;Acc:HGNC:7579] | 8463 | -0.191 | -0.2819 | No |
| 75 | TJP1 | tight junction protein 1 [Source:HGNC Symbol;Acc:HGNC:11827] | 8523 | -0.195 | -0.2809 | No |
| 76 | PRKCG | protein kinase C gamma [Source:HGNC Symbol;Acc:HGNC:9402] | 8582 | -0.200 | -0.2796 | No |
| 77 | RHOA | ras homolog family member A [Source:HGNC Symbol;Acc:HGNC:667] | 8601 | -0.201 | -0.2752 | No |
| 78 | GNAI2 | G protein subunit alpha i2 [Source:HGNC Symbol;Acc:HGNC:4385] | 8686 | -0.208 | -0.2757 | No |
| 79 | CLDN11 | claudin 11 [Source:HGNC Symbol;Acc:HGNC:8514] | 8817 | -0.219 | -0.2795 | No |
| 80 | VAPA | VAMP associated protein A [Source:HGNC Symbol;Acc:HGNC:12648] | 8854 | -0.221 | -0.2759 | No |
| 81 | CLDN14 | claudin 14 [Source:HGNC Symbol;Acc:HGNC:2035] | 9033 | -0.234 | -0.2830 | No |
| 82 | MRAS | muscle RAS oncogene homolog [Source:HGNC Symbol;Acc:HGNC:7227] | 9158 | -0.244 | -0.2856 | No |
| 83 | RRAS2 | RAS related 2 [Source:HGNC Symbol;Acc:HGNC:17271] | 9165 | -0.245 | -0.2790 | No |
| 84 | CLDN9 | claudin 9 [Source:HGNC Symbol;Acc:HGNC:2051] | 9203 | -0.248 | -0.2747 | No |
| 85 | JAM3 | junctional adhesion molecule 3 [Source:HGNC Symbol;Acc:HGNC:15532] | 9376 | -0.262 | -0.2805 | No |
| 86 | PARD6A | par-6 family cell polarity regulator alpha [Source:HGNC Symbol;Acc:HGNC:15943] | 9703 | -0.292 | -0.2976 | No |
| 87 | F11R | F11 receptor [Source:HGNC Symbol;Acc:HGNC:14685] | 9765 | -0.297 | -0.2937 | No |
| 88 | TJP2 | tight junction protein 2 [Source:HGNC Symbol;Acc:HGNC:11828] | 9772 | -0.298 | -0.2855 | No |
| 89 | CLDN5 | claudin 5 [Source:HGNC Symbol;Acc:HGNC:2047] | 9816 | -0.302 | -0.2801 | No |
| 90 | TJP3 | tight junction protein 3 [Source:HGNC Symbol;Acc:HGNC:11829] | 10260 | -0.347 | -0.3047 | No |
| 91 | PPP2R1A | protein phosphatase 2 scaffold subunit Aalpha [Source:HGNC Symbol;Acc:HGNC:9302] | 10395 | -0.360 | -0.3047 | Yes |
| 92 | ACTN4 | actinin alpha 4 [Source:HGNC Symbol;Acc:HGNC:166] | 10472 | -0.370 | -0.2999 | Yes |
| 93 | MYL12B | myosin light chain 12B [Source:HGNC Symbol;Acc:HGNC:29827] | 10505 | -0.374 | -0.2915 | Yes |
| 94 | KRAS | "KRAS proto-oncogene, GTPase [Source:HGNC Symbol;Acc:HGNC:6407]" | 10544 | -0.377 | -0.2836 | Yes |
| 95 | PPP2R1B | protein phosphatase 2 scaffold subunit Abeta [Source:HGNC Symbol;Acc:HGNC:9303] | 10578 | -0.382 | -0.2751 | Yes |
| 96 | RAB13 | "RAB13, member RAS oncogene family [Source:HGNC Symbol;Acc:HGNC:9762]" | 10961 | -0.425 | -0.2926 | Yes |
| 97 | CLDN16 | claudin 16 [Source:HGNC Symbol;Acc:HGNC:2037] | 11104 | -0.444 | -0.2908 | Yes |
| 98 | CLDN7 | claudin 7 [Source:HGNC Symbol;Acc:HGNC:2049] | 11178 | -0.454 | -0.2833 | Yes |
| 99 | CTNNA2 | catenin alpha 2 [Source:HGNC Symbol;Acc:HGNC:2510] | 11342 | -0.477 | -0.2822 | Yes |
| 100 | MAP3K20 | mitogen-activated protein kinase kinase kinase 20 [Source:HGNC Symbol;Acc:HGNC:17797] | 11475 | -0.498 | -0.2781 | Yes |
| 101 | CTTN | cortactin [Source:HGNC Symbol;Acc:HGNC:3338] | 11677 | -0.528 | -0.2785 | Yes |
| 102 | SYMPK | symplekin [Source:HGNC Symbol;Acc:HGNC:22935] | 11757 | -0.542 | -0.2689 | Yes |
| 103 | MAGI1 | "membrane associated guanylate kinase, WW and PDZ domain containing 1 [Source:HGNC Symbol;Acc:HGNC:946]" | 11770 | -0.545 | -0.2540 | Yes |
| 104 | PTEN | phosphatase and tensin homolog [Source:HGNC Symbol;Acc:HGNC:9588] | 11836 | -0.556 | -0.2430 | Yes |
| 105 | CLDN15 | claudin 15 [Source:HGNC Symbol;Acc:HGNC:2036] | 11849 | -0.559 | -0.2277 | Yes |
| 106 | CSNK2A1 | casein kinase 2 alpha 1 [Source:HGNC Symbol;Acc:HGNC:2457] | 11895 | -0.570 | -0.2146 | Yes |
| 107 | YBX3 | Y-box binding protein 3 [Source:HGNC Symbol;Acc:HGNC:2428] | 12024 | -0.599 | -0.2073 | Yes |
| 108 | CLDN18 | claudin 18 [Source:HGNC Symbol;Acc:HGNC:2039] | 12254 | -0.662 | -0.2060 | Yes |
| 109 | ASH1L | ASH1 like histone lysine methyltransferase [Source:HGNC Symbol;Acc:HGNC:19088] | 12405 | -0.708 | -0.1972 | Yes |
| 110 | PRKCI | protein kinase C iota [Source:HGNC Symbol;Acc:HGNC:9404] | 12444 | -0.723 | -0.1791 | Yes |
| 111 | YES1 | "YES proto-oncogene 1, Src family tyrosine kinase [Source:HGNC Symbol;Acc:HGNC:12841]" | 12450 | -0.725 | -0.1585 | Yes |
| 112 | PARD3 | par-3 family cell polarity regulator [Source:HGNC Symbol;Acc:HGNC:16051] | 12489 | -0.743 | -0.1399 | Yes |
| 113 | ACTG1 | actin gamma 1 [Source:HGNC Symbol;Acc:HGNC:144] | 12613 | -0.806 | -0.1261 | Yes |
| 114 | CLDN3 | claudin 3 [Source:HGNC Symbol;Acc:HGNC:2045] | 12615 | -0.807 | -0.1027 | Yes |
| 115 | CDC42 | cell division cycle 42 [Source:HGNC Symbol;Acc:HGNC:1736] | 12681 | -0.852 | -0.0831 | Yes |
| 116 | ACTB | actin beta [Source:HGNC Symbol;Acc:HGNC:132] | 12757 | -0.920 | -0.0622 | Yes |
| 117 | CTNNB1 | catenin beta 1 [Source:HGNC Symbol;Acc:HGNC:2514] | 12866 | -1.119 | -0.0382 | Yes |
| 118 | AKT2 | AKT serine/threonine kinase 2 [Source:HGNC Symbol;Acc:HGNC:392] | 12896 | -1.404 | 0.0003 | Yes |
Table: GSEA details [plain text format]

  

Fig 2: KEGG\_TIGHT\_JUNCTION      
 Blue-Pink O' Gram in the Space of the Analyzed GeneSet

  

Fig 3: KEGG\_TIGHT\_JUNCTION: Random ES distribution      
 Gene set null distribution of ES for **KEGG\_TIGHT\_JUNCTION**
